# Pluripotency markers are differentially induced by IGF1 and bFGF in cells from patients’ lesions of large/giant congenital melanocytic nevi

**DOI:** 10.1186/s40364-018-0152-9

**Published:** 2019-01-14

**Authors:** Dipanjan Basu, Cláudia M. Salgado, Janki R. Patel, Joie Zabec, Ryan M. Hoehl, Bruce Bauer, Miguel Reyes-Múgica

**Affiliations:** 1 Department of Pathology, Children’s Hospital of Pittsburgh, University of Pittsburgh, Pittsburgh, PA 15224 USA; 2 Dietrich School of Arts and Sciences, University of Pittsburgh, Pittsburgh, PA USA; 3 Division of Plastic and Reconstructive Surgery, North Shore University Health System, Northbrook, IL USA

**Keywords:** Melanocytes, Giant congenital nevi, Melanoma

## Abstract

Factors regulating transcription of pluripotency genes in congenital nevo-melanocytes are not known. Nevo-melanocytes belong somewhere in-between the ends of a spectrum where the normal epidermal melanocyte represents one end and a melanoma cell with multiple genetic abnormalities represents the other. Cells from large/giant congenital nevi (L/GCMN), unlike normal melanocytes, grow colonies on soft agar and express pluripotency markers, similar to melanoma cells. In this study normal melanocytes, SKMEL28 melanoma cells and nevo-melanocytes isolated from three L/GCMN patients were exposed to niche factors bFGF and IGF1 in vitro at physiological doses, and expression of a panel of pluripotency markers was determined by RT-PCR. While normal melanocytes did not show any significant transcriptional change in the genes studied, bFGF induced transcription of Sox2 and Bmi1 in melanoma cells. Patients’ cells showed differential expression, with Sox10 being common to C76N and PD1N, while only Sox2 and Bmi1 were upregulated in C139N. IGF1 on the other hand induced unique sets of genes in each individual sample. We conclude that expression of pluripotency genes in L/GCMN cells is affected by niche factors bFGF and IGF1; however, each individual growth factor induced a unique set of genes in a patient’s cells.

**Electronic supplementary material:**

The online version of this article (10.1186/s40364-018-0152-9) contains supplementary material, which is available to authorized users.

## Main text

Congenital melanocytic nevi are characterized by proliferations of pigmented cells of neural crest origin, usually present at birth or appearing shortly thereafter. They have been traditionally classified based on size. An infrequent but well-known complication is the occurrence of melanoma. Large/giant congenital nevi (L/GCMN) are recently assessed to have an overall 2% risk of melanoma transformation [1, 2]. Although, cells from L/GCMN are known to harbor a post-zygotic somatic mutation in *NRAS* or *BRAF*, detailed molecular characteristics of these neoplastic cells are not completely understood. Recent reports underscore the proliferative, tumorigenic and clonogenic potential of these cells [3, 4]. We have shown that nevomelanocytes from Neurocutaneous melanocytosis (NCM), one of the feared complications associated with L/GCMN, can be grown as *Nevospheres* in vitro and be used as experimental models for drug testing [5]. Sphere formation in an anchorage independent culture system is classically accepted as one of the functional characteristics of stem cells that express pluripotency genes or stemness marker genes, such as Bmi1, Oct4, Sox2 etc. Clonogenic cells from L/GCMN were found to express Oct4, Nestin and Sox10 [3]. However, the external factors regulating expression of stemness genes inside these cells are not known. Here, we report the role of IGF1 and bFGF on the expression of a panel of stemness marker genes in cells isolated from lesions of three L/GCMN patients.

As (i) sphere forming ability is considered a functional characteristic of tumor stem cells, and (ii) expression of pluripotency marker genes characterize stemness, knowing the factors regulating transcription of such genes would be critical to understanding how stemness is maintained in these cells. Growth factors bFGF and IGF1, two important niche factors in skin, were critical to grow nevomelanocytic cells in culture [5, 6]. Therefore, we asked whether these niche factors have a regulatory role on the transcription of some of the pluripotency marker genes in L/GCMN cells. To facilitate comparison, we chose normal newborn melanocytes and SKMEL28 melanoma cell line as controls.

### Patients and samples

De-identified patients’ samples were prospectively collected with consent following guidelines approved by University of Pittsburgh Institutional Review Board. Clinical information of the patient’s lesion is described in Additional file 1: Table S1 (Main nevus size categorization was according to Krengel et al. [7]) Nevomelanocytes from three different donors were cultured in Medium 254 from ThermoFisher Scientific with supplements as previously described [5]. Normal human epidermal melanocytes were cultured using the same medium. Melanoma cell line SKMEL28 was cultured following instructions from ATCC using EMEM with 10% fetal bovine serum. Experiments were set up for each cell type in serum/supplement free conditions with or without the stated growth factors at the indicated dose. Cells were harvested after 72 h. and total RNA was extracted.

### RNA extraction and PCR

Total RNA was extracted using Qiagen RNeasy kit. Q-PCR was conducted using primers for Sox2, Sox10, Pax3, MITF, Bmi1, Nestin and Oct4 genes. Validated primer sets were obtained from Realtimeprimers.com. Data were analyzed using GraphPad prism software. Statistical analysis was performed using SPSS software (IBM SPSS version 25). Each experiment was repeated at least three times.

## Results

**Fig. 1:**
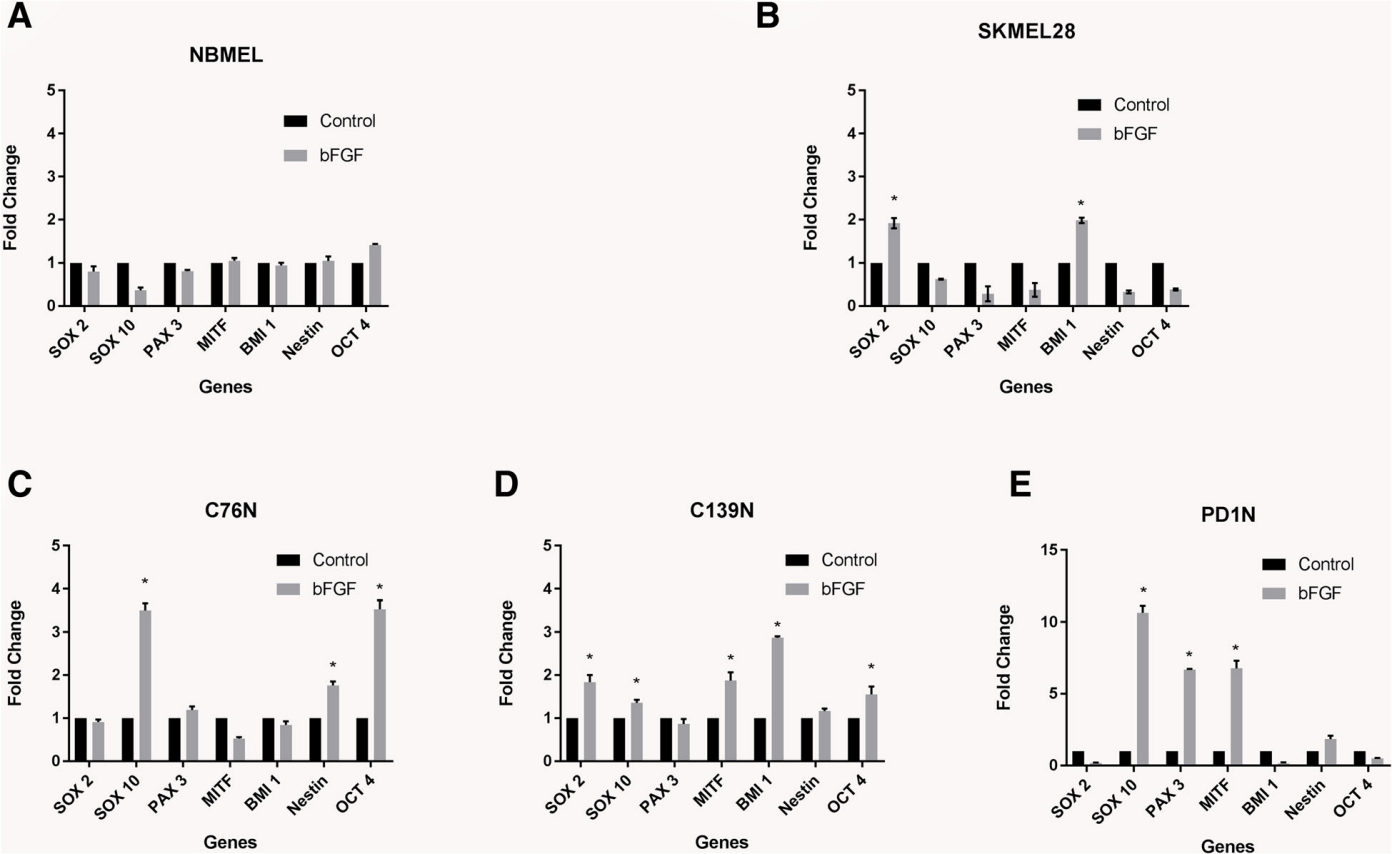
Effect of bFGF treatment on the transcription of stemness marker genes. Cells were treated or not with 3 ng/ml bFGF after 24 h in the serum/supplement-free medium. Cells were harvested in RNA extraction buffer 48 h after the treatment. qPCR performed with GAPDH serving as internal housekeeping control. Fold change in gene expression was calculated by ΔΔCt method. Fold change in untreated controls was considered as 1 by convention. Relative fold changes compared between untreated (control) and treated cells were plotted for all the indicated cell types. Statistical analyses were performed for relative expression between cell types. Significance (*p* < 0.05) was indicated by asterisk after performing one way ANOVA. **a** NBMEL: newborn melanocytes; **b**. SKMEL28: melanoma; **c** patient C76N; **d** patient 139N; **e** patient PD1N

**Fig. 2:**
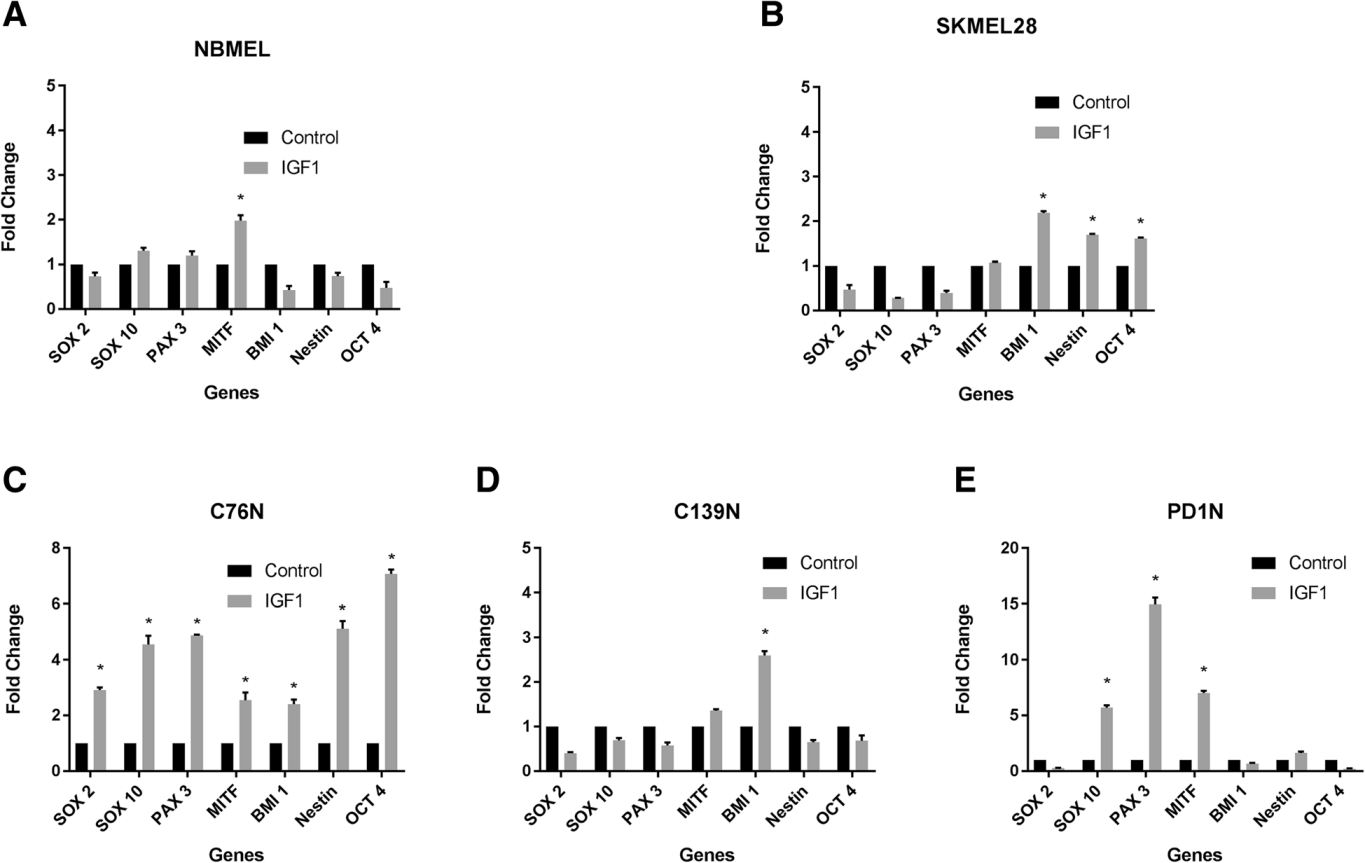
Effect of IGF1 treatment on the transcription of stemness marker genes. Cells were treated or not with 1 μg/ml IGF1 after 24 h in the serum/supplement-free medium. Cells were harvested in RNA extraction buffer 48 h after the treatment. qPCR performed with GAPDH serving as internal housekeeping control. Fold change in gene expression was calculated by ΔΔCt method. Fold change in untreated controls was considered as 1 by convention. Relative fold changes compared between untreated (control) and treated cells were plotted for all the indicated cell types. Significance (*p* < 0.05) was indicated by asterisk after performing one way ANOVA. **a** NBMEL: newborn melanocytes; **b**. SKMEL28: melanoma; **c** patient C76N; **d** patient 139N; **e** patient PD1N

A panel of stemness marker genes was selected based on previous reports of neural crest, melanocyte specific and general stemness marker genes in published literature [3, 8, 9]. Newborn melanocytes (NBMEL) did not show any change in the transcription of the genes studied, with or without bFGF (Fig. 1a). In SKMEL28 melanoma cells, only Sox2 and Bmi1 showed a 2-fold upregulation when treated with bFGF (Fig. 1b). Bmi1 was also upregulated approximately 2-fold in SKMEL28 cells upon IGF1 treatment along with Nestin and Oct4 which are only slightly induced (Fig. 2b). In normal melanocytes only MITF expression was induced by IGF1 suggesting that this transcription factor responsible for maintaining the melanocytic phenotype, is regulated by IGF1 in normal melanocytes (Fig. 2a). When compared to NBMEL, nevomelanocytes from patient C76N showed upregulation in most of the stemness marker genes’ transcription, as determined by quantitative real time PCR, when treated with IGF1 (Fig. 2c.). However, bFGF was able to induce upregulation in the transcription of only Sox10 and Oct4 genes at a level of statistical significance (Fig. 1c.). In cells from patient C139N, significant upregulation was noted in Bmi1, with nearly a 2-fold increase in Sox2, MITF and Oct4 genes upon bFGF treatment (Fig. 1d), while IGF1 only induced Bmi1 in the same cells (Fig. 2d.). The extent of upregulation was notably higher in cells from patient PD1N. The only genes affected were Sox10, Pax3 and MITF, but were induced up to about 6–10 fold with bFGF treatment (Fig. 1e.). Pax3 transcription was increased even further with IGF1 treatment (Fig. 2e.). Taken together, bFGF could induce Sox2 in melanoma cells which remained largely unaffected in NBMEL and the nevomelanocytes with the exception being C139N. Basic Fibroblast Growth Factor also induced Bmi1 in C139N similar to melanoma cells but not in the others. IGF1 on the other hand induced MITF in NBMEL and nevomelanocytes but not in melanoma cells. Two of the nevomelanocytic cell lines responded highly to IGF1 treatment by upregulating most of the stemness marker genes studied, which is significantly different than NBMEL and melanoma cells. Statistical analyses of the gene expression differences are provided in Additional file 1: Table S1 and Additional file 2: Table S2.

## Conclusion

One major difference between normal human skin melanocytes and human primary or metastatic melanoma cells is that skin melanocytes do not grow colonies in soft agar, while melanoma cells have clonogenic efficiencies ranging from 0 to 63% in soft agar [10]. Nevomelanocytes are oncogenically transformed neoplastic cells that are capable of clonogenic growth in culture – a property similar to the melanoma cells. The extent of genetic alterations in nevomelanocytes is not known completely. The majority of L/GCMN lesions reported in literature shows a single oncogenic mutation (in either NRAS or BRAF) which is thought to be sufficient to drive tumorigenesis. Compared to that, most melanoma cell lines have accumulated additional genetic alterations over and above the driver mutations making them more aggressively proliferating. In this context, it is important to note that the melanoma cell line SKMEL28 used in this study has been previously reported to form clonogenic colonies in anchorage independent soft agar cultures [11] and express stemness markers Oct3/4 and Nanog [12]. However, in this study, SKMEL28 did not show induction of stemness genes by bFGF or IGF1. It is possible that the stemness genes investigated in this study, including Oct3/4 are not regulated by either bFGF or IGF1 in SKMEL28. In comparison, nevomelanocytes isolated from all three patients responded to bFGF and IGF1 treatment by upregulating pluripotency genes. The extent of upregulation and the pattern of expression varied between the patient samples and the nature of the growth factor. It is possible that each growth factor induces separate signaling pathways leading to expression of a unique set of genes related to stemness. It remains to be investigated if the nature of oncogenic mutation has a bearing on the mechanism of transcriptional regulation of pluripotency genes. Normal newborn melanocytes are grown in adherent culture and they do not form colonies under non-adherent conditions.

## Additional files

Additional file 1: **Table S1**. Difference in gene expression between the normal melanocytes and CMN cells after treatment with bFGF/IGF1 (DOCX 21 kb)

Additional file 2: **Table S2**. Difference in gene expression between the skin melanoma cells and CMN cell after treatment with bFGF/IGF-1 (DOCX 17 kb)

Additional file 3: **Table S3** (DOCX 15 kb)

## Abbreviations

ATCC: American type culture collection
bFGF: Basic fibroblast growth factor
IGF1: Insulin like growth factor 1
L/GCMN: Large/giant congenital melanocytic nevi
MITF: Micropthalmia Transcription factor
NBMEL: Newborn melanocyte
NCM: Neurocutaneous melanocytosis
RTPCR: Real time polymerase chain reaction

## References

[1] Krengel S, Hauschild A, Schäfer T (2006). Melanoma risk in congenital melanocytic naevi: a systematic review. Br J Dermatol.

[2] Vourc'h-Jourdain M, Martin L, Barbarot S (2013). Large congenital melanocytic nevi: Therapeutic management and melanoma risk: A systematic review. J Am Acad Dermatol.

[3] Charbel C, Fontaine RH, Kadlub N, Coulomb-L'Hermine A, Rouille T, How-Kit A, Moguelet P, Tost J, Picard A, Aractingi S, Guegan S (2015). Clonogenic cell subpopulations maintain congenital melanocytic nevi. J Invest Dermatol.

[4] Guégan S, Kadlub N, Picard A, Rouillé T, Charbel C, Coulomb-L'Hermine A, How-Kit A, Fraitag S, Aractingi S, Fontaine RH (2016). Varying proliferative and clonogenic potential in NRAS-mutated congenital melanocytic nevi according to size. Exp Dermatol.

[5] Basu D, Salgado CM, Bauer BS, Johnson D, Rundell V, Nikiforova M, Khakoo Y, Gunwaldt LJ, Panigrahy A, Reyes-Múgica M (2016). Nevospheres from neurocutaneous melanocytosis cells show reduced viability when treated with specific inhibitors of NRAS signaling pathway. Neuro-Oncology.

[6] Mancianti ML, Gyorfi T, Shih IM, Valyi-Nagy I, Levengood G, Menssen HD, Halpern AC, Elder DE, Herlyn M (1993). Growth regulation of cultured human nevus cells. J Invest Dermatol.

[7] Krengel S, Scope A, Dusza SW, Vonthein R, Marghoob AA (2013). New recommendations for the categorization of cutaneous features of congenital melanocytic nevi. J Am Acad Dermatol.

[8] Kerosuo L, Nie S, Bajpai R, Bronner Marianne E (2015). Crestospheres: long-term maintenance of multipotent, Premigratory neural crest stem cells. Stem Cell Reports.

[9] Kreso A, van Galen P, Pedley NM, Lima-Fernandes E, Frelin C, Davis T, Cao L, Baiazitov R, Du W, Sydorenko N (2014). Self-renewal as a therapeutic target in human colorectal cancer. Nat Med.

[10] Melber K, Zhu G, Diamond L (1989). SV40-transfected human melanocyte sensitivity to growth inhibition by the Phorbol Ester 12-<em>O</em>-Tetradecanoylphorbol-13-acetate. Cancer Res.

[11] Woodard J, Platanias LC (2010). AMP-activated kinase (AMPK)-generated signals in malignant melanoma cell growth and survival. Biochem Biophys Res Commun.

[12] Dorris ER, Blackshields G, Sommerville G, Alhashemi M, Dias A, McEneaney V, Smyth P, O'Leary JJ, Sheils O (2016). Pluripotency markers are differentially induced by MEK inhibition in thyroid and melanoma BRAFV600E cell lines. Cancer Biol Ther.

