# Supplementary material for: Pluripotency markers are differentially induced by IGF1 and bFGF in cells from patients’ lesions of large/giant congenital melanocytic nevi
Source: Biomark Res. 2019 Jan 14;7:2. doi: 10.1186/s40364-018-0152-9 (PMC6332894; doi:10.1186/s40364-018-0152-9)
Supplement: Supplementary file 1 — Table S1. Difference in gene expression between the normal melanocytes and CMN cells after treatment with bFGF/IGF1 (DOCX 21 kb) [file 40364_2018_152_MOESM1_ESM.docx]

**Additional file 1: Table S1**

Difference in gene expression between the normal melanocytes and CMN cells after treatment with bFGF/IGF1

| Gene /growth factor (I) cell (J) cell | Mean Difference (I-J) | Sig. | 95% CI  Lower Bound | 95% CI Upper Bound |
| --- | --- | --- | --- | --- |
| Sox2 / FGF NBMEL C76N  C139N  PD1N | -0.107  -1.035*  0.638* | 0.766  <0.001  <0.001 | -0.407  -1.335  0.338 | 0.193  -0.735  0.938 |
| Sox10 / FGF NBMEL C76N  C139N  PD1N | -3.139*  -0.994*  -10.279* | <0.001  0.003  <0.001 | -3.767  -1.622  -10.907 | -2.511  -0.366  -9.651 |
| Pax3 / FGF NBMEL C76N  C139N  PD1N | -0.382*  -0.054  -5.853* | 0.027  0.980  <0.001 | -0.720  -0.393  -6.191 | -0.043  0.284  -5.515 |
| MITF / FGF NBMEL C76N  C139N  PD1N | 0.525  -0.823*  -5.553* | 0.096  0.017  <0.001 | -0.084  -1.489  -6.162 | 1.133  -0.156  -4.945 |
| Bmi1 / FGF NBMEL C76N  C139N  PD1N | 0.096  -1.926*  0.797* | 0.421  <0.001  <0.001 | -0.079  -2.102  0.622 | 0.272  -1.751  0.973 |
| Nestin / FGF NBMEL C76N  C139N  PD1N | -0.710*  -0.115  -0.799* | <0.001  0.780  <0.001 | -1.039  -0.444  -1.129 | -0.380  0.215  -0.470 |
| Oct4 / FGF NBMEL C76N  C139N  PD1N | -2.120*  -0.144  0.915* | <0.001  0.716  0.001 | -2.512  -0.536  0.477 | -1.728  0.248  1.354 |
| Sox2 / IGF NBMEL C76N  C139N  PD1N | -2.177*  0.333*  0.466* | <0.001  0.002  <0.001 | -2.376  0.133  0.267 | -1.977  0.532  0.666 |
| Sox10 / IGF NBMEL C76N  C139N  PD1N | -3.250*  0.610*  -4.389* | <0.001  0.011  <0.001 | -3.718  0.143  -4.856 | -2.783  1.077  -3.922 |
| Pax3 / IGF NBMEL C76N  C139N  PD1N | -3.684*  0.618  -13.76* | <0.001  0.127  <0.001 | -4.441  -0.139  -14.513 | -2.927  1.375  -12.999 |
| MITF / IGF NBMEL C76N  C139N  PD1N | -0.563*  0.624*  -5.021* | 0.014  0.007  <0.001 | -1.014  0.174  -5.472 | -0.112  1.075  -4.571 |
| Bmi1 / IGf NBMEL C76N  C139N  PD1N | -1.974*  -2.171*  -0.254 | <0.001  <0.001  0.075 | -2.252  -2.482  -0.532 | -1.697  -1.861  0.023 |
| Nestin / IGF NBMEL C76N  C139N  PD1N | -4.376*  0.069  -0.879* | <0.001  0.974  <0.001 | -4.764  -0.319  -1.267 | -3.988  0.457  -0.491 |
| Oct4 / IGF NBMEL C76N  C139N  PD1N | -6.595*  -0.202  0.271 | <0.001  0.180  0.054 | -6.903  -0.477  -0.005 | -6.288  0.073  0.546 |

NBMEL: Normal epidermal melanocytes

ANOVA one way, Tukey post hoc test. *p<0.05
