# Supplementary material for: Pluripotency markers are differentially induced by IGF1 and bFGF in cells from patients’ lesions of large/giant congenital melanocytic nevi
Source: Biomark Res. 2019 Jan 14;7:2. doi: 10.1186/s40364-018-0152-9 (PMC6332894; doi:10.1186/s40364-018-0152-9)
Supplement: Supplementary file 2 — Table S2. Difference in gene expression between the skin melanoma cells and CMN cell after treatment with bFGF/IGF-1 (DOCX 17 kb) [file 40364_2018_152_MOESM2_ESM.docx]

**Additional file 2: Table S2**

Difference in gene expression between the skin melanoma cells and CMN cell after treatment with bFGF/IGF-1

| Dependent Variable (I) cell (J) cell | Mean Difference (I-J) | Sig. | 95% CI  Lower Bound | 95% CI Upper Bound |
| --- | --- | --- | --- | --- |
| Sox2 / FGF SKMEL C76N  C139N  PD1N | 1.009*  0.081  1.754* | <0.001  0.894  <0.001 | 0.709  -0.289  1.454 | 1.309  0.381  2.054 |
| Sox10 / FGF SKMEL C76N  C139N  PD1N | -2.878*  -0.732*  -10.018* | <0.001  0.021  <0.001 | -3.505  -1.360  -10.645 | -2.250  -0.105  -9.390 |
| Pax3 / FGF SKMEL C76N  C139N  PD1N | -0.382*  -0.054  -5.853* | 0.027  0.980  <0.001 | -0.719  -0.393  -6.191 | -0.044  0.284  -5.515 |
| MITF/ FGF SKMEL C76N  C139N  PD1N | -0.151  -1.498*  -6.229* | 0.867  <0.001  <0.001 | -0.695  -2.106  -6.773 | 0.394  -0.890  -5.685 |
| Bmi1 / FGF SKMEL C76N  C139N  PD1N | 1.137*  -0.886*  1.837* | <0.001  <0.001  <0.001 | 0.961  -1.062  1.662 | 1.312  -0.711  2.013 |
| Nestin / FGF SKMEL C76N  C139N  PD1N | -1.431*  -0.835*  -1.520* | <0.001  <0.001  <0.001 | -1.760  -1.165  -1.850 | -1.101  -0.506  -1.191 |
| Oct4 / FGF SKMEL C76N  C139N  PD1N | -3.145*  -1.169*  -0.110 | <0.001  <0.001  0.926 | -3.584  -1.608  -0.591 | -2.707  -0.731  0.370 |
| Sox2 / IGF SKMEL C76N  C139N  PD1N | -2.444*  0.066  0.199* | <0.001  0.807  0.050 | -2.643  -0.133  0.001 | -2.244  0.265  0.399 |
| Sox10 / IGF SKMEL C76N  C139N  PD1N | -4.269*  -0.409  -5.407* | <0.001  0.094  <0.001 | -4.736  -0.876  -5.875 | -3.802  0.058  -4.941 |
| Pax3 / IGF SKMEL C76N  C139N  PD1N | -4.482*  -0.180  -14.554* | <0.001  0.930  <0.001 | -5.239  -0.937  -15.311 | -3.725  0.577  -13.797 |
| MITF / IGF SKMEL C76N  C139N  PD1N | -1.471*  -0.284  -5.929* | <0.001  0.302  <0.001 | -1.922  -0.734  -6.380 | -1.021  0.167  -5.479 |
| Bmi1/ IGf SKMEL C76N  C139N  PD1N | -0.223  -0.420*  1.497* | 0.131  0.009  <0.001 | -0.500  -0.730  1.220 | 0.055  -0.110  1.775 |
| Nestin / IGF SKMEL C76N  C139N  PD1N | -3.422*  1.023*  0.075 | <0.001  <0.001  0.965 | -3.810  0.635  -0.313 | -3.034  1.411  0.463 |
| Oct4 / IGF SKMEL C76N  C139N  PD1N | -5.462*  0.931*  1.404* | <0.001  <0.001  <0.001 | -5.769  0.656  1.129 | -5.155  1.206  1.679 |

SKMEL: SKMEL28 skin melanoma cell line

ANOVA one way, Tukey post hoc test. *p<0.05
