# Supplementary material for: Pluripotency markers are differentially induced by IGF1 and bFGF in cells from patients’ lesions of large/giant congenital melanocytic nevi
Source: Biomark Res. 2019 Jan 14;7:2. doi: 10.1186/s40364-018-0152-9 (PMC6332894; doi:10.1186/s40364-018-0152-9)
Supplement: Supplementary file 3 — Table S3 (DOCX 15 kb) [file 40364_2018_152_MOESM3_ESM.docx]

Additional file 3: Table S3

| Patient ID | Age | Sex | Main Nevus Size* | Location |
| --- | --- | --- | --- | --- |
| C76N | 1 yr 9 mts | Male | G2 | Skin |
| C139N | 1 yr 2 mts | Female | M1 | Skin |
| PD1N | 2 yr 5 mts | Male | G2 | Skin |

*Main nevus size characterization according to Krengel et al [7]
